# Functional genomics reveals that *Clostridium difficile* Spo0A coordinates sporulation, virulence and metabolism

**DOI:** 10.1186/1471-2164-15-160

**Published:** 2014-02-25

**Authors:** Laura J Pettit, Hilary P Browne, Lu Yu, Wiep Klaas Smits, Robert P Fagan, Lars Barquist, Melissa J Martin, David Goulding, Sylvia H Duncan, Harry J Flint, Gordon Dougan, Jyoti S Choudhary, Trevor D Lawley

**Affiliations:** 1 Wellcome Trust Sanger Institute, Hinxton, UK; 2 Department of Medical Microbiology, Leiden University Medical Centre, Leiden, The Netherlands; 3 Department of Molecular Biology and Biotechnology, University of Sheffield, Sheffield, UK; 4 European Molecular Biology Laboratory, European Bioinformatics Institute, Hinxton, UK; 5 Rowett Institute of Nutrition and Health, University of Aberdeen, Aberdeen, UK; 6 Bacterial Pathogenesis Laboratory, Wellcome Trust Sanger Institute, Hinxton, Cambridgeshire CB10 1SA, UK

**Keywords:** *Clostridium difficile*, Spo0A, RNAseq, Proteomics, Sporulation, Virulence, Metabolism, Butyrate, Transmission, Genome annotation

## Abstract

**Background:**

*Clostridium difficile* is an anaerobic, Gram-positive bacterium that can reside as a commensal within the intestinal microbiota of healthy individuals or cause life-threatening antibiotic-associated diarrhea in immunocompromised hosts. *C. difficile* can also form highly resistant spores that are excreted facilitating host-to-host transmission. The *C. difficile spo0A* gene encodes a highly conserved transcriptional regulator of sporulation that is required for relapsing disease and transmission in mice.

**Results:**

Here we describe a genome-wide approach using a combined transcriptomic and proteomic analysis to identify Spo0A regulated genes. Our results validate Spo0A as a positive regulator of putative and novel sporulation genes as well as components of the mature spore proteome. We also show that Spo0A regulates a number of virulence-associated factors such as flagella and metabolic pathways including glucose fermentation leading to butyrate production.

**Conclusions:**

The *C. difficile spo0A* gene is a global transcriptional regulator that controls diverse sporulation, virulence and metabolic phenotypes coordinating pathogen adaptation to a wide range of host interactions. Additionally, the rich breadth of functional data allowed us to significantly update the annotation of the *C. difficile* 630 reference genome which will facilitate basic and applied research on this emerging pathogen.

## Background

*Clostridium difficile* has emerged over the past decade to become the most common cause of infectious antibiotic-associated diarrhea within healthcare systems worldwide [1]. This Gram-positive, anaerobic bacterium commonly resides asymptomatically in healthy individuals who can serve as a transmission reservoir within a hospital setting [2]. The emergence of *C. difficile* virulence is linked to the acquisition of multiple resistance determinants to commonly used antibiotics [3-6] allowing this pathogen to thrive in the intestines of patients following antibiotic treatment [7]. *C. difficile* can produce a number of potent virulence-associated factors that contribute to intestinal colonization and disease [8], and facilitate the establishment of a pathological imbalance within the resident microbiota [9]. Unlike many other healthcare pathogens, *C. difficile* produces highly resistant and transmissible spores and, as a consequence, creates significant challenges to infection control and environmental decontamination protocols [10].

Sporulation is a complex developmental program leading to the generation of metabolically dormant spores from vegetative cells [11]. Spo0A is a transcription factor that is active in the early stages of sporulation in *C. difficile* and other sporulating bacteria [12]. Orthologues of Spo0A are encoded by a variety of the Firmicutes [13], including *Bacillus* and *Clostridium*, and the genetic inactivation of this gene leads to a non-sporulating phenotype [14,15]. In the well studied *Clostridium* and *Bacillus* organisms, many of the pleiotropic effects of Spo0A are due to indirect regulation via the transition state regulator AbrB [16-20]. However, *C. difficile* does not encode an AbrB orthologue highlighting differences in Spo0A activity between *C. difficile* and other *Clostridium* and *Bacillus* species studied to date [5,21]. *C. difficile* Spo0A binds directly to DNA upstream of several early sporulation genes [21] but this transcriptional regulator may also control other processes not obviously associated with sporulation. For example, in *C. difficile* Spo0A has also been implicated in controlling toxin gene expression [22] and disease in mice [15,23] and biofilm formation *in vitro*[24,25].

Consequently, we performed a genome-wide analysis to define Spo0A regulated genes within *C. difficile* using a combined transcriptomic and proteomic approach. Our analysis demonstrates that the *C. difficile spo0A* gene encodes a global transcriptional regulator that coordinates an array of phenotypes associated with host colonization and transmission. The rich breadth of our functional data allowed us to make significant updates to the annotation of the *C. difficile* 630 reference genome.

## Results

### *C. difficile* growth dynamics *in vitro*

**Figure 1:**
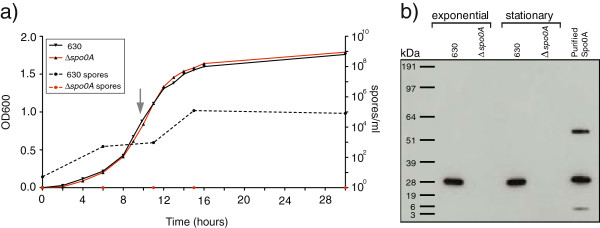
**Growth and sporulation kinetics of *C. difficile *630 derivatives in broth cultures. a)** Growth curves of the *C. difficile* 630*∆erm* parental strain and its *spo0A* isogenic mutant. Shown are the OD_600_ measurements on the left y-axis and ethanol-resistant spore-derived CFUs on the right y-axis. The gray vertical arrow indicates that sampling point. **b)** Western blot analysis demonstrating that Spo0A is expressed under exponential and stationary growth stages by the parental *C. difficile* 630*∆erm* strain but not the isogenic *spo0A* mutant. Spo0A-his6 protein was purified protein as described before [21].

We and others [14,15,23] have previously demonstrated that *C. difficile* 630*Δerm* produces spores and that an isogenic *spo0A*::*ermB* mutant does not, but that sporulation can be restored by expressing the *spo0A* gene *in trans*[23]. Here we confirm this phenotype in Wilson’s broth supplemented with glucose [26] and show that *C. difficile* 630*Δerm* and the *spo0A* mutant displayed comparable growth kinetics under these culture conditions with shaking (Figure 1a) which allows for more reproducible growth compared to cultures grown statically [27]. Interestingly, we found that the sporulation program was active primarily during exponential phase, leading to an increase in the formation of ethanol resistant spores at the transition into stationary growth phase. We observed no discernable increase in spores during stationary phase (Figure 1a). During mid-exponential stage approximately 1 in 100,000 cells (10^3^ spores/ml) and during late-exponential approximately 1 in 3,000 cells (10^5^ spores/ml) were ethanol-resistant spores (Figure 1a). The growth conditions used in our study are not optimized for sporulation, as we wished to obtain a broad picture of Spo0A dependent transcriptional effects, rather than identifying the Spo0A-dependent sporulation program. As a result, the observed spore levels are lower than achieved with other growth conditions [27-29].

Western blot analysis using *C. difficile* Spo0A-specific antibodies demonstrated that Spo0A was produced during both exponential and stationary growth by *C. difficile* 630*Δerm* but not a *spo0A* mutant derivative (Figure 1b). Given our interest in defining the genes controlled by *C. difficile* Spo0A and the potential links between sporulation and other phenotypes we chose to focus on mid-exponentially growing *C. difficile* (vertical gray arrow in Figure 1a) for subsequent experiments.

### Functional enrichment of the *C. difficile* 630 genome annotation with transcriptomics and proteomics

To survey and compare the global transcriptomes of *C. difficile* 630*Δerm* and the *spo0A* mutant we performed high-density, strand-specific cDNA sequencing (RNAseq) of RNA extracted from exponentially growing cultures and mapped the sequence data to the *C. difficile* 630 genome [5,30]. Next we normalised the mean abundance of reads per gene (Additional file 1) and identified 321 genes that were differentially expressed (*P*-adjusted value < 0.01) in the *spo0A* mutant, of which 164 were upregulated and 157 were downregulated compared to *C. difficile* 630*Δerm* (Additional file 2). Genes whose expression was influenced by Spo0A (either positively or negatively) were evenly distributed around the genome and were encoded on both the forward and reverse strands (data not shown).

**Figure 2:**
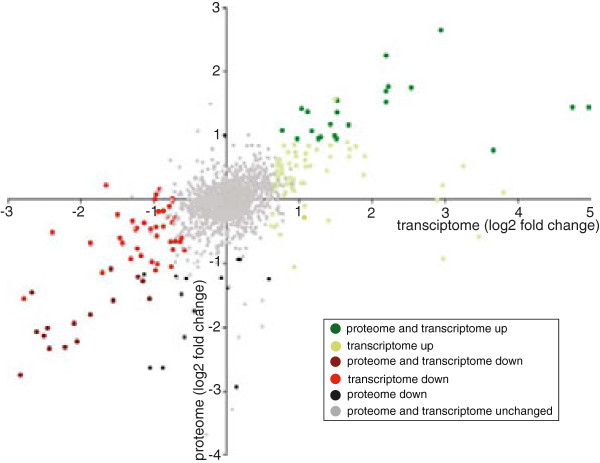
**Correlation between transcriptomic and proteomic datasets of genes differentially expressed in the *C. difficile spo0A *mutant compared to the parental *C. difficile *630*Δerm *strain.** The degree of correlation are plotted as log2 fold change for all gene products that had been quantified in both proteome (y-axis) and RNASeq (x-axis) analyses. Significantly (p-adj < 0.01) disregulated proteins or transcripts are shown with red/brown (upregulated) and light green/dark green (downregulated) symbols. Protein groups that were positive in Significance B test of at least two biological replicates are depicted as significant (dark colours). See methods for analysis details. Note that no proteins were upregulated without being upregulated at the transcript level (“proteome up”).

We also performed comparative proteomic analysis on the same *C. difficile* cultures to complement the RNAseq dataset. Proteins were extracted from the same samples used for RNA analysis and separated on SDS-PAGE followed by in-gel digestion and peptide extraction. To compare protein levels, the generated peptides were labeled with dimethyl stable isotope labels and mixed prior to mass spectrometry analysis. Peptide identification and quantitation were assigned using MaxQuant software. Using a 1% false discovery rate (FDR) we identified polypeptide products corresponding to 1000 genes. Notably, the proteomic analysis showed better coverage for abundant proteins and a higher proportion of cytoplasmic compared to membrane or secreted proteins (Additional file 2). We found 123 proteins that were differentially regulated by a log2 fold change, of which 75 proteins were decreased and 48 increased in relative abundance in the *spo0A* mutant derivative compared to *C. difficile* 630*Δerm*. A strong correlation was observed (Figure 2) between the transcriptomic and proteomic datasets. A detailed break down of each dataset into function class as well as the intersection of the trancriptomic and proteomic analysis datasets are given as a searchable excel file in Additional file 2.

Our combined transcriptomic and proteomic dataset comparing the 630*Δerm* and the *spo0A* mutant derivatives provided a unique opportunity to enrich the current genome annotation of the reference *C. difficile* 630, particularly for those genes that were previously annotated as “conserved hypothetical” based on *in silico* predictions [5,30]. This study represents the largest functional analysis of the reference *C. difficile* 630 genome since its creation in 2006 [5] and builds upon previous annotation updates [30] by validation of the implicated genes that is not possible by *in silico* methods.

The improved annotation described here is primarily based on the presence of differentially expressed genes in the RNAseq and proteomic datasets (this study) or the mature spore proteome [27] datasets (Additional file 2). For those genes implicated in the experimental dataset that did not have a known functional product, *in silico* annotation was undertaken to predict a function. This utilised searches on nucleotide and amino acid sequences of homologous genes and prediction of protein family and domain sites with the Pfam database [31] and Prosite database [32] in addition to identifying subcellular localisation of proteins using SignalP [33] and THMMM [34]. This process was also complemented by literature searches relating to the functional characterisation of the genes and their products in question. A feature of any annotated genome is that it is current only at the time it was created. In light of this *in silico* annotation as described above was also performed on all genes without a known product that were not present in the experimental dataset.

**Figure 3:**
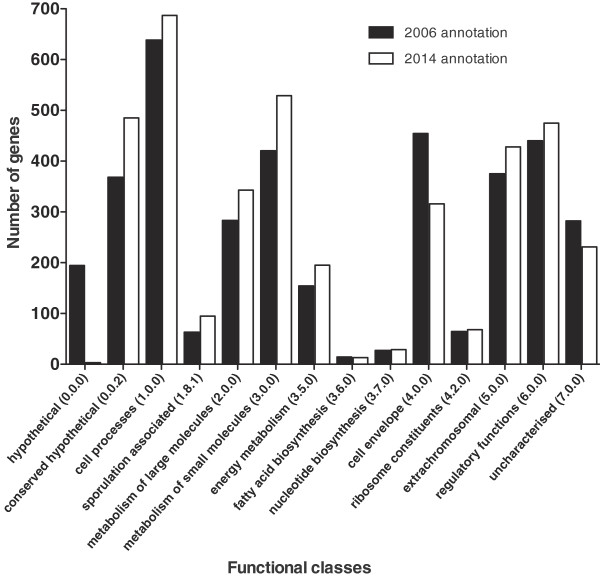
**Updated functional class annotation of *C. difficile *630 genome compared to original 2006 genome annotation **[5]**.** Genes are classified according to functional classes for the original 2006 annotation (black bars) compared to this study (white bars). Functional classes are described in more detail in Additional file 2.

The initial annotation of the *C. difficile* 630 genome characterised genes by functional classes adapted from the Riley class system [35] (Additional file 2). For brevity some classes have been collapsed into broader functional descriptions. The number of genes in most functional classes has increased since that original annotation [5] (Figure 3). The notable exceptions are the classes ‘fatty acid biosynthesis’ (class 3.6.0), a decrease of 1, and ‘cell envelope’ (class 4.0.0), a decrease of 138. Genes previously in class 4.0.0 have now transferred to a wide range of different functional classes including ‘cell processes’ (class 1.0.0), ‘sporulation’ (class 1.8.1), ‘extrachromosomal’ (class 5.0.0) and ‘conserved hypothetical’ (class 0.0.2) reflecting transitions between functional classes based on more accurate annotation tools and increasing numbers of *C. difficile* genomic studies.

Correspondingly the functional classes characterising genes of an unknown or a putative function i.e. ‘hypothetical’ (class 0.0.0), ‘conserved hypothetical’ (class 0.0.2) and ‘uncharacterised’ (class 7.0.0), have all decreased in number except for class 0.0.2. However 92 of these genes were not identified in the initial 2006 annotation (Figure 3) and a further 133 were initially part of class 0.0.0 and have been moved to class 0.0.2 representing annotation studies on subsequent *C. difficile* strains. As a result, there are now only 3 genes in class 0.0.0 (CD2933, CD2947A and CD3148).

In total 662 genes were differentially expressed in the *spo0A* RNAseq, *spo0A* proteome or the mature spore proteome datasets representing 17% of the entire *C. difficile* 630 gene content. 11 of the 662 differentially expressed genes that previously were classified as 0.0.2 or 7.0.0 now have a known function, these include 4 spore coat associated genes: CD1433 (*cotE*), CD1511 (*cotB*), CD1567 (*cotG*) and CD1613 (*cotA*) [36,37]. The presence of 68 genes in the experimental dataset that previously resided in class 0.0.2 are now updated to class 7.0.0 confirming their expression. Interestingly, 6 genes that were previously classified as pseudogenes due to the presence of frameshifts or mobile genetic elements have been changed to functioning genes based on their presence in the experimental dataset. 2 of these (CD0816 and CD1718) are interrupted by IStrons. The ability of the IStron to be excised from the mRNA transcript and not interfere with gene functionality [38,39] is corroborated by the presence of these genes in the experimental dataset.

*In silico* annotation of genes in classes 0.0.0, 0.0.2 and 7.0.0 that were not present in the experimental dataset also resulted in considerable improvements to the elucidation of gene products. 24 genes previously contained in these classes are now in a known functional class. The classification of 9 more genes has improved from class 0.0.2 to class 7.0.0. In addition to this a further 5 genes (CD0606, CD1499, CD1570, CD1820 and CD2553), previously classified as pseudogenes due to been interrupted by IStrons have been restored to functional genes. All of these improvements are representative of a broad range of functional classes.

Now 3409 out of the 3897 genes in the *C. difficile* 630 genome have either a known or putative function or experimental evidence confirming the gene is expressed. Other significant improvements to the annotation include the re-classification of *sigK* (CD1230) as a functional gene; the presence of the *skin* prophage-like element which interrupts the gene is not detrimental to gene functionality due to controlled excision [40].

In addition to identifying gene products, gene nomenclatures have been updated where possible. Gene nomenclatures for 26 of the cell wall proteins (*cwp*) [41] have been added as well as the cationic peptide resistance cluster *cprA,B,C* and its regulators *cprR* and *cprK*[42,43]. Gene names for the extra cytoplasmic sigma factors *csfU,V,T* and the associated anti-sigma factors *rsiU,V,T* which are involved in sensing and responding to extracellular stresses [44] have also been added. Finally, duplicate gene names have been resolved where appropriate by the addition of a number or letter.

The annotation has been updated on the main genomic sequence repositories (accession no. AM180355) and the embl file can also be accessed at http://www.sanger.ac.uk/resources/downloads/bacteria/clostridium-difficile.html which contains all of the functional class annotations described here. Subsequent analysis in this manuscript was performed using the newly annotated *C. difficile* 630 genome.

### Genome-wide functional classification of the Spo0A regulated genes

**Figure 4:**
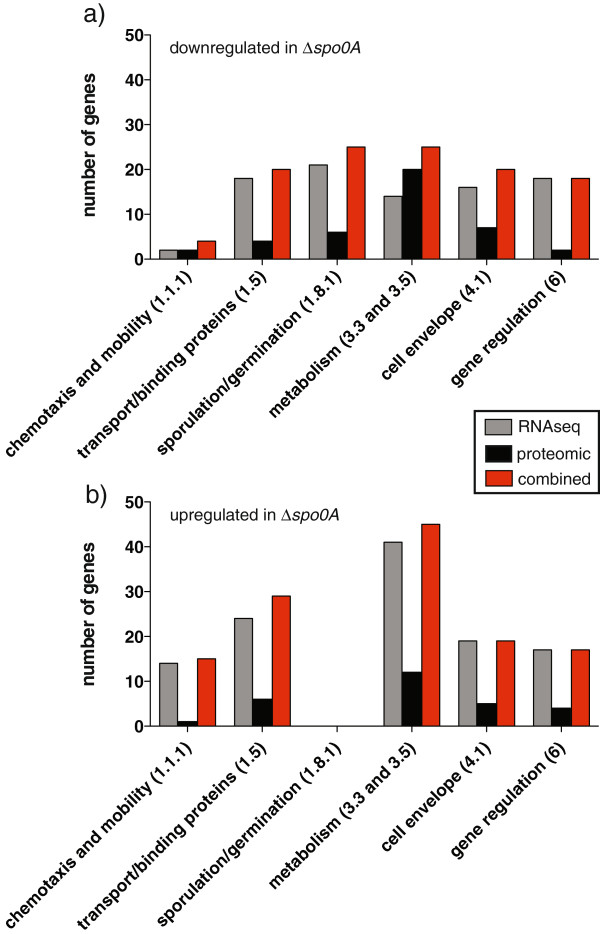
**Functional classification of *C. difficile *Spo0A regulated genes.** Enriched functional gene classes of genes **(a)** downregulated or **(b)** upregulated in the *C. difficile spo0A* mutant. The number of genes present in each functional class based on RNAseq (grey) and proteomics (black) analysis. The combined RNAseq and proteomics (red) represents unique genes found in each functional class. Transcripts and proteins assigned to functional classes are given in parenthesis and are based on the updated *C. difficile* 630 annotation presented in this study (Additional file 2).

The differentially expressed genes identified by transcriptomics and proteomics were assigned into functional classes based on a modified version of the Riley system [5,35] (Figure 4). Our analysis indicated that Spo0A regulates genes representing a broad array of functional classes (Additional file 2), although there was an enrichment of certain classes (Figure 4a and b). For example, of the 96 genes in the sporulation/germination functional class, 25 genes were positively regulated by Spo0A, that is, their expression was attenuated in the *spo0A* mutant, whereas no genes in this class were relatively upregulated (Figure 4a and b). Gene classes comparatively downregulated in the *spo0A* mutant include those assigned to the functional classes transport/binding (20), metabolism (24), cell-envelope architecture (20) and gene regulation (19) (Figure 4a). In contrast, genes linked to the functional classes chemotaxis/mobility (15), transport/binding (28), metabolism (45), cell-envelope architecture (20) and gene regulation (18) were upregulated in the *spo0A* mutant relative to the parental strain (Figure 4b).

Several genes that were previously shown to harbor a consensus Spo0A binding site upstream of their coding sequences [21] were differentially regulated in the *spo0A* mutant at the transcript or protein level. For instance, Spo0A binds to the upstream region of *ssuA*, which shows significantly lower RNA and protein levels in the *spo0A* mutant compared to wild type (Additional file 2). Similarly, the putative direct target *lplA* shows increased levels of RNA and protein. Thus, these genes are likely direct targets of Spo0A in *C. difficile*. We found that many genes with altered transcript levels do not contain a consensus Spo0A binding motif [21]. These may either reflect regulation through a non-consensus Spo0A-binding motif, or indirect regulation. It is noteworthy that several putative transcriptional regulators show changes in transcript levels (Additional file 3) and strong Spo0A dependent regulation without the presence of a clear Spo0A binding site has previously been noted for *Clostridium acetobutylicum*[45].

Overall, these data indicate the *C. difficile* Spo0A directly and indirectly regulates a diverse set of genes potentially linked to different phenotypes beyond sporulation.

### Spo0A positively regulates the sporulation cascade

**Figure 5:**
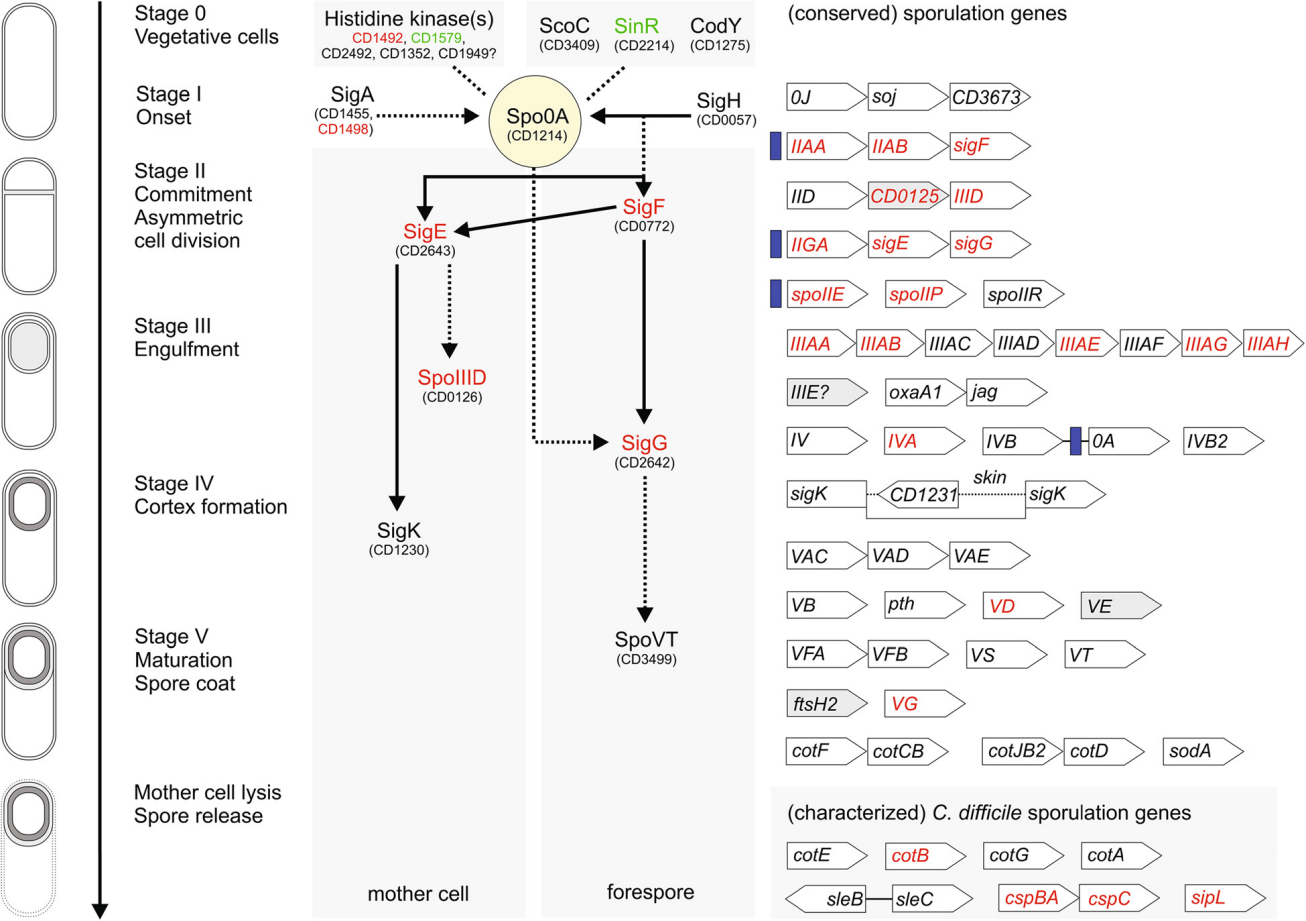
**Proposed sporulation cascade for *Clostridium difficile *630.** Names of genes and proteins are derived from updated genome annotation (Additional file 2). Solid arrows in the putative regulatory cascade of *C. difficile* indicate confirmed interactions [15,21], whereas dotted arrows indicate uncharacterized or postulated interactions. Operon structure or genomic region of (conserved) sporulation genes is given when relevant, and *in vitro* confirmed Spo0A binding sites are indicated with vertical blue bars. Genes are roughly aligned with the stage of sporulation at which they act, except for *C. difficile* sporulation genes that are not conserved in *B. subtilis*. When gene names start with *spo*, this has been omitted for clarity. CD3673 encodes a protein with high similarity to Spo0J. CD0125 encodes a protein with homology to *B. subtilis spoIIQ. spoIV* encodes a homolog of the *B. subtilis* YqfD protein. *sigK* is known as *spoIIIC* (N-terminal part) and *spoIVCB* (C-terminal part) in *B. subtilis* and is interrupted by a *skin* element. CD1231 encodes the recombinase in *skin* and is annotated as *spoIVCA* in *B. subtilis. oxaA1* is known as *spoIIIJ* in *B. subtilis. pth* is known as *spoVC* in *B. subtilis. spoVE* is an FtsW-like protein and is sometimes annotated as such in *C. difficile*. The product of *ftsH2* is the closest homolog of SpoVK of *B. subtilis. C. difficile* 630 encodes several SpoIIIE/FtsK like proteins. If and which one is associated with sporulation is unknown. *cotF*/*cotCB* and *cotJB2/cotD* are homologs of the *B. subtilis* genes *cotJB* and *cotJC*. SleB is also known as PrsW [44]. CspBA is a serine protease. Proteins from this family in *B. subtilis* are not directly identified as sporulation specific. CspC is a germination receptor [48]. *sipL* was hypothesized to encode a functional substitute for *B. subtilis* SpoVID [49]. CD1613 (*cotA*), CD1511 (*cotB*), CD1433 (*cotE*), CD1567 (*cotG*) have been given a *cot* alias in a recent study [37]. For the genes in grey the identification as homologs of the *B. subtilis* sporulation gene is tentative. Green colors indicate upregulated and red colors indicate downregulated in a *spo0A* mutant compared to the parental *C. difficile* 630*∆erm* strain at the transcriptome or proteome level in this study. Recently, the transcription of many genes - including most of the genes from this scheme - was identified as dependent on sporulation specific sigma factors [28,29,50].

Our analysis of the *C. difficile* Spo0A-asssociated sporulation pathway was guided by an abundance of knowledge available for the sporulation pathway in *B. subtilis*[46]. *C. difficile* 630 encodes a number of orthologues for genes such as CodY, SinR and ScoC that regulate the earliest stages of sporulation in *B. subtilis* upstream of Spo0A*.* Neither *codY* nor *scoC* genes are under the control of Spo0A (Figure 5). However, the *sinR* gene was upregulated in the *C. difficile spo0A* mutant, indicating negative regulation by Spo0A, similar to *B. subtilis*[47] (Additional file 2). *C. difficile* 630 does not harbour orthologues of the Spo0A phosphorelay proteins of *B. subtilis* that lead to Spo0A phosphorylation. However, others have suggested that one or more orphan histidine kinases of *C. difficile* can directly or indirectly affect Spo0A activation [15] and two of these genes encoding potential kinases, CD1492 and CD1579, are differentially expressed in the *spo0A* mutant versus wild type cells (Figure 5) (Additional file 2).

Phosphorylation of Spo0A leads to the activation of a sigma factor cascade that acts in both mother cell and forespore (Figure 5) and many of the early stage *B. subtilis* sporulation proteins are highly conserved in *C. difficile* 630 genome, including the sporulation specific sigma factors SigH, SigF, SigG, SigE, SigA2 and SigK [11]. We found that Spo0A influenced the expression of the *sigE*, *sigG*, *sigF* and *sigA2* genes that were relatively under represented in the *spo0A* mutant. Taken together, these data suggest that in *C. difficile* Spo0A positively controls the expression of SigF, SigG, SigA2 and SigE during the early stages of sporulation (Figure 5).

Our data indicates that the role of Spo0A in regulating the formation of endospores is relatively conserved between *B. subtilis* and *C. difficile*. However, based on genomic and proteomic comparisons the later stages (cortex, coat and release; stages IV and V: reviewed in [11]) of sporulation appear to be less conserved. We found the three transcriptional units encoding early sporulation genes previously predicted to be direct targets of Spo0A in *C. difficile*[21], *spoIIAA*-*AB*-*AC*/*sigF*, *spoIIGA*-*sigG*-*sigE* and *spoIIE*, were under represented in the *spo0A* mutant. The list of significantly affected genes also includes SigE-dependent genes, such as *spoIIID* and the *spoIIIAA*-*AH* operon. Of all of these stage II and III genes in the Spo0A regulon, only SigG is found in the mature spore (Additional file 2).

It should be noted that our analysis did not identify many of the conserved later-stage IV and V genes [11] as differentially expressed in the *spo0A* mutant, likely due to the growth conditions. Many of these are controlled by the late sporulation sigma factor SigK, and consistent with this we observed no effect of a *spo0A* mutation on *sigK* transcription under our experimental conditions. The exceptions are *spoIVA*, *spoVD, cspBA-cspC* and *sipL* which are under control of SigE [51]. We also identified the genes encoding the subtilisin like protease CspBA [52], the germinant receptor CspC [48], and the spore morphogenetic protein SipL [49] as differentially expressed in the *spo0A* mutant.

### Spo0A regulates colonization and virulence genes

Our analysis revealed that distinct functional classes linked to *C. difficile* virulence and colonization are under control of Spo0A. Consistent with our previous studies [23], the toxin gene *tcdA* was significantly upregulated in the *C. difficile spo0A* mutant (Additional file 2). We could not identify a consensus ‘0A’ binding box upstream of the *tcdA* start site and *in vitro* binding assays did not indicate that *tcdA* was a direct target of Spo0A [21]. Thus, Spo0A appears to indirectly control *tcdA* gene expression, perhaps via one of the many other factors that control toxin gene expression [53-56]. Our analyses were carried out on cells in exponential growth phase, where toxin expression is lower compared to stationary growth phase [57]. This may in part explain the fact that the *tcdB* gene (which is expressed at much lower levels than *tcdA*[56,58] was not identified as differentially expressed.

Our data suggests that Spo0A exerts significant control over the *C. difficile* cell envelope architecture and associated structures (Figure 4a and b), several of which are implicated in intestinal adherence, colonisation or subversion of the hosts’ immune system. For example, six genes encoding surface proteins showed altered transcription in the *spo0A* mutant; *cwp10*, CD2797 and CD3246 are upregulated in the *spo0A* mutant whereas *cwp19*, *cwp27* and *cwp29* are all downregulated. Two of these, CD2797 and CD3246 possess adherence-associated domains while the remaining four (*cwp10*, *cwp19*, *cwp27* and *cwp29*) encode members of the cell wall protein family [41]. Several genes implicated in remodeling of the cell wall are also regulated by Spo0A. For example, *uppS1* encodes an undecaprenyl-pryophosphate synthase responsible for synthesizing a cell wall carbohydrate lipid precursor, and is downregulated in the *spo0A* mutant. Two genes in the *dlt* alanylation operon, *dltA* and *dltB*, were significantly upregulated in the *spo0A* mutant. These later two genes are located within a four-gene operon involved in the esterification of teichoic acid or lipoteichoic acid with D-alanine, conferring resistance to cationic antimicrobial peptides [59].

In *C. difficile* 630, components of the flagellar assembly apparatus are encoded by two loci that are divided by an inter-flagellar locus that has been implicated in flagellin glycosylation. Fourteen genes encoding flagellar proteins (*fliC, fliE, fliF, fliG, fliH, fliI, fliJ, fliK, fliW, fliZ, flbD, flgB, flgD* and *motB*) and the four transcripts (CD0241-CD0244) that constitute the inter-flagellar glycosylation locus [60] were relatively upregulated in the *spo0A* mutant at the mRNA level (Additional file 2). However, the differences in expression of the flagella structural genes were not reflected in the proteomic data and this is likely due to loss of the flagella from bacterial cells during sample preparation.

We could not identify a consensus Spo0A binding motifs immediately upstream of the Spo0A-affected cell membrane/wall associated genes, with the exception of putative CD0241-0244 operon [21]. This indicates that the majority of the effects of Spo0A on this class of genes are either mediated by degenerate motifs or indirectly via one or more of the Spo0A-affected transcriptional regulators (Additional file 3).

**Figure 6:**
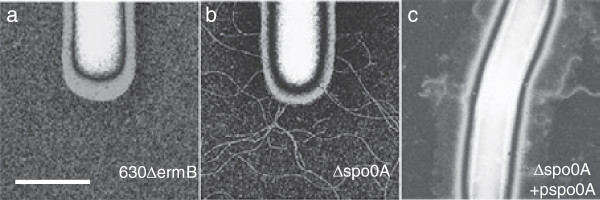
**Spo0A is a negative regulator of *C. difficile *flagellar synthesis.** Representative transmission electron micrographs of negatively stained *C. difficile* 630∆*erm* and derivatives demonstrating **a)** no observable flagella on the parental 630∆*erm* strain but **b)** hyper-flagellation in the *spo0A* mutant derivative. Genetic complementation of the **c)***spo0A* mutation greatly reduced flagella levels but did not eliminate their production. Scale bar represents 1 micron.

To validate these observations we examined *C. difficile* 630*Δerm* and the isogenic *spo0A* mutant for the presence of peritrichous flagella by negative staining and TEM. We consistently observed no cell-anchored flagella on the parental *C. difficile* 630*Δerm* (Figure 6a). However, the *spo0A* mutant displayed a striking hyper-flagellate phenotype with an average of 8.1 flagella/bacterium (n = 210; average length 8.8 μm and average diameter 15.2 nm) (Figure 6b). Complementation with a plasmid-borne copy of *spo0A* partially restored the phenotype and reduced the average number of flagella to 4.3 per bacterium (n = 240; average length 3.5 μm and average diameter 14.6 nm) (Figure 6c). Together these results demonstrate a role of Spo0A in negatively controlling *C. difficile* flagella production.

### *C. difficile* Spo0A is a positive regulator of butyrate biosynthesis

**Figure 7:**
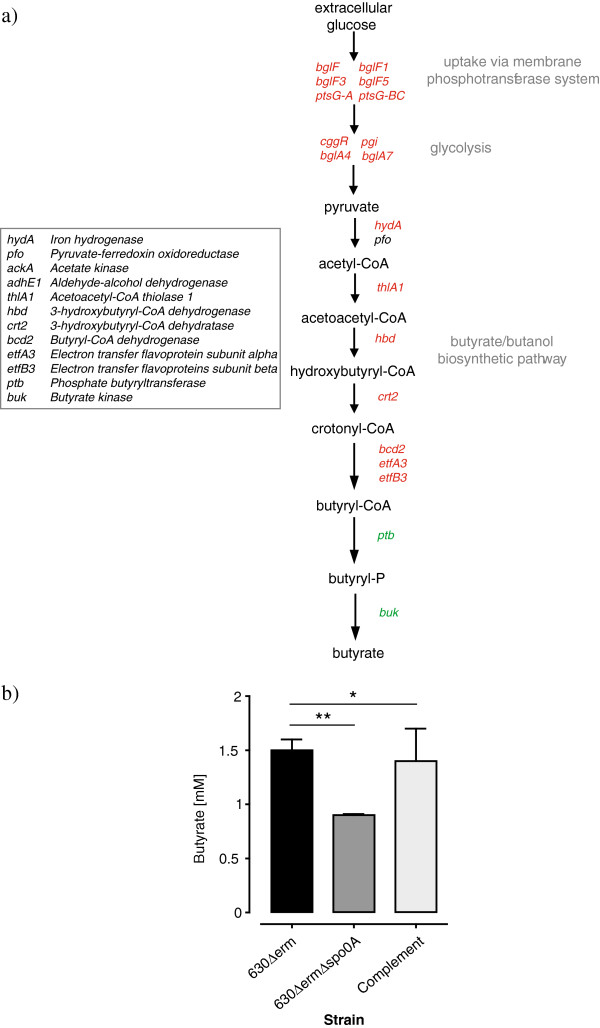
**Spo0A positively regulates glucose fermentation pathways and butyrate production in *C. difficile. *a)** Proposed biochemical pathway for glucose uptake and fermentation leading to the production of butyrate. Genes in red are downregulated in the *C. difficile spo0A* mutant and genes in green are upregulated in the *C. difficile spo0A* mutant. Genes in black are not impacted by the *spo0A* mutation. **b)** Levels of butyrate from supernatants of *C. difficile* strains during exponential growth. Analysis was performed in triplicate and levels compared using a Student’s *T* test; **, P = 0.0005; *, P = 0.6.

Spo0A positively regulates several regulatory genes predicted to be involved in carbohydrate uptake and metabolism (Additional file 2). For example, 3 separate *bglG*-type genes involved in carbohydrate sensing and transcriptional anti-termination are downregulated in a *spo0A* mutant, and 7 transcriptional regulators are downregulated in the *spo0A* mutant, most of which are predicted to respond to nutrients and extracellular cues (Additional file 2). Further, several membrane-associated transporters of the phosphotransferase system (PTS) with predicted specificity for beta-glucoside (*bglF*-type) are downregulated in the *spo0A* mutant (Figure 7a and Additional file 2). Key genes from the glycolysis pathway that convert glucose to pyruvate were downregulated in the *spo0A* mutant, including the glucose-6-phosphate isomerase (*pgi*), the central glycolytic genes regulator (*cggR*) and putative 6-phospho-alpha- and beta-glucosidases (*bglA4* and *bglA7,* respectively).

Glucose fermentation can lead to the production of a variety of metabolic by-products including butyrate (glucose is the only carbohydrate in the growth medium). Interestingly, we found that the entire butyrate production operon [61] was downregulated in the *C. difficile spo0A* mutant at both the mRNA and protein levels (Additional file 2), including butyryl-CoA dehydrogenase (*bcd2*), electron transfer flavoprotein β-subunit (*etfB3*), electron transfer flavoprotein α-subunit (*etfA3*), 3-hydroxybutyryl-CoA dehydratase (*crt2)*, 3-hydroxybutyryl-CoA dehydrogenase (*hbd)* and acetyl-CoA acetyltransferase (*thlA1)*. Figure 7 illustrates a proposed biochemical pathway for glucose uptake and fermentation highlighting in red those genes under positive control of Spo0A. Key components of this butyrate production pathway are also present in the *C. difficile* 630 spore proteome [27], suggesting a possible association between butyrate and spore formation and/or germination in *C. difficile*. None of the genes listed above harbor a consensus Spo0A binding site in their immediate upstream region. Interestingly, *ptb* and *buk*, encoding a phosphate butyryltransferase and a butyrate kinase, respectively, were upregulated in the *spo0A* mutant at the mRNA level. These two genes are potentially directly regulated by Spo0A; the upstream region of *ptb* contains a Spo0A box [21] and the *buk* gene likely forms an operon with *ptb*.

To determine which of these transcriptional responses dominates the butyrate biosynthetic pathway we measured butyrate from exponentially growing *C. difficile* 630*Δerm* and the *spo0A* mutant. We found that the *C. difficile spo0A* mutant produced significantly less butyrate than the parental 630*Δerm* (*P* = 0.0005; Figure 7). Moreover, the *spo0A* mutant complemented *in trans* with a plasmid borne *spo0A* gene produced levels of butyrate that were statistically comparable with the 630*Δerm* parental strain (*P* = 0.6; Figure 7). Thus, overall *C. difficile* Spo0A positively controls butyrate production, likely via an indirect mechanism.

## Discussion

*C. difficile* Spo0A is generally considered a transcriptional regulator of sporulation although recent reports have suggested additional roles in toxin gene regulation *in vitro*[15,23] and intestinal colonization and disease in mice [23]. Here we demonstrate an unappreciated role for *C. difficile* Spo0A as a global transcriptional regulator of colonization, virulence, sporulation and metabolic phenotypes. Thus, Spo0A influences all aspects of the *C. difficile* infection cycle allowing the bacterium to adapt to changing environments experienced during infection and host-transmission. The wide array of phenotypic defects in the *C. difficile spo0A* mutant would explain our previous results demonstrating that the *C. difficile spo0A* gene is required for persistent colonization and disease in mice [23].

Spo0A is a transcriptional regulator that is activated via phosphorylation from a cognate membrane-associated sensor histidine kinase(s) in response to an environmental cue(s). Underwood *et al.*[15] demonstrated that the histidine kinase CD2492 can potentially serve as a phosphorylation donor for *C. difficile* Spo0A and proposed that other histidine kinases (i.e. CD1492 and CD1579) may also interact with Spo0A suggesting multiple environmental inputs. The environmental cues that lead to *C. difficile* Spo0A phosphorylation remain unknown but we predict such cues to be mainly present within the mammalian intestinal tract when *C. difficile* is metabolically active. Further, we propose such environmental cues influence an array of physiological states and phenotypes during the complete *C. difficile* lifecycle since Spo0A is present in the mature spore [27] and during all stages of vegetative growth [21].

*C. difficile* sporulation in the laboratory seems to occur asynchronously [28,49,50]. Though our sampling timepoint likely covers multiple growth stages, we cannot exclude the possibility that specific early or late timepoint-specific effects of Spo0A are missed in our analyses. Indeed, as a result of our choice of timepoint, late sporulation genes are underrepresented in our study. However, several studies have specifically addressed the role of Spo0A and the downstream sporulation specific sigma factors in the sporulation program [28,29,50]. Our genome and proteome analysis provides an excellent starting point to discriminate genes under direct or indirect control of Spo0A in *C. difficile*. We find that Spo0A affects the expression of several regulators (Additional file 3), suggesting that many of the observed effects may be indirect. Future research will focus on defining those environmental cues and associated histidine kinases that lead to Spo0A phosphorylation and the downstream genes that are under direct control of Spo0A-P to delineate the *C. difficile* Spo0A regulon.

Our study defined Spo0A-dependent gene expression patterns and phenotypes that allow us to speculate on *C. difficile’s* lifestyle within the intestinal tract. For example, Spo0A negatively regulates the sporulation cascade and surface proteins proposed to promote host interactions (i.e. CD2797 and CD3246) in addition to negatively regulating virulence factor expression (flagella and Toxin A). Perhaps during sporulation *C. difficile* downregulates some virulence and motility functions to avoid host surveillance while adhering to the mucosal surface. Indeed, we routinely observe *C. difficile* sporulating on the mucosal surface within the murine intestinal tract [62,63]. Spo0A also coordinates shifts in nutrient transporters and metabolic pathways perhaps in response to the available nutrients on the mucosal surface such as complex and simple carbohydrates within the mucus layer overlying the intestinal epithelial cells [64]. Spo0A also positively regulates butyrate production, a significant energy source for intestinal epithelial cells [65], potentially representing a metabolic link between *C. difficile* and the host to promote mucosal adherence. It is of note that several of the genes and programs identified in our study were also found to be Spo0A dependent in *C. acetobutylicum*[45,66], suggesting that these are conserved and physiologically relevant patterns.

Even though we linked *C. difficile* Spo0A to distinct physiological states and phenotypes the vast majority of genes misregulated in a *C. difficile spo0A* mutant are of putative or hypothetical function due to the lack of knowledge about *C. difficile* biology and host interactions. The rich breadth of transcriptome and proteome data provided a unique opportunity to perform a significant functional annotation to the *C. difficile* 630 reference genome. With the recent description of genetic mutagenesis methods [14,52] and murine infection models [62,67,68] the updated genome annotation will facilitate studies into *C. difficile* colonization, disease and transmission.

## Conclusions

The *C. difficile* spo0A gene is a global transcriptional regulator that controls diverse sporulation, virulence and metabolic phenotypes coordinating pathogen adaptation to a wide range of host interactions. Additionally, the rich breadth of functional data allowed us to significantly update the annotation of the *C. difficile* 630 reference genome which will facilitate basic and applied research on this emerging pathogen.

## Methods

### Bacterial strains and growth conditions

*C. difficile* strains 630*Δerm,* 630*Δerm spo0A*::*ermB* (named the *spo0A* mutant in this manuscript) and 630*Δerm spo0A*::*ermB* + p*spo0A* were previously described [23]. *C. difficile* was grown at 37°C under anaerobic conditions in a MACS MG-500 anaerobic workstation (Don Whitley Scientific). *C. difficile* was routinely cultured in Wilson’s broth plus 1% glucose with agitation (80 rpm) or on CCEY agar (Bioconnections) supplemented with cycloserine (250 μg/ml; Bioconnections), cefoxitin (8 μg/ml; Bioconnections) and 0.1% taurocholate (Sigma Aldrich) for 24 to 48 hours. For the enumeration of spores, *C. difficile* cultures were mixed with 100% ethanol (1:1 ratio) for 1 h at room temperature to kill vegetative cells, pelleted, washed in PBS and cultured as above.

### Western blotting

Proteins resolved by SDS-PAGE were electrophoretically transferred to a nitrocellulose membrane at 30 V for 1 h. Protein transfer was visualised by staining in Ponseau-S Red (Sigma Aldrich) for 2 min, and membranes were blocked in blocking buffer (5% milk powder in 0.1% PBS-T) for 1 h at room temperature. Membranes were then probed with a Spo0A primary antibody (1/10,000) [21] overnight at 4°C, washed, and detected with an appropriate HRP-conjugated secondary antibody (1/10,000) for 1 h at room temperature. Proteins were revealed by chemiluminescence detection according to the Amersham ECL system (GE Healthcare), as per the manufacturer’s instructions.

### RNA preparation and cDNA synthesis

Total RNA isolation and cDNA synthesis was performed as previously described [69]. Briefly, three biological replicates of *C. difficile* culture (~10^10^ total cells) from exponentially growing cells were harvested in RNAProtect (Qiagen) according to the manufacturer’s protocol. Total RNA was extracted by chemical and mechanical lysis using a FastRNA Pro Blue Kit (MP Biomedicals) and FastPrep ribolyser, according to the manufacturer’s recommendations. Total RNA was purified using the SV RNA Isolation Purification Kit (Promega) according to the manufacturer’s instructions. Genomic DNA was removed from total RNA samples using one treatment of Turbo DNase (Ambion) according to the manufacturer’s recommendations. RNA quantification and integrity was determined using both a ND-1000 (NanoDrop Technologies) and 2100 Bioanalyser (Agilent Technologies). Samples were screened for the presence of genomic DNA using primer pairs CD1498 F: GATTGCAGATGCATGTGGTT and CD1498 R: TTGGAGAGCAAGAACAGCAA, CD1455 F: GATGCAGAGGCAATTTCACA and CD1455 R: GCTAGAAGGATGCACGAAGG, CD0011F: CCAGCTTTGCAACACCAACT and CD0011 R: GGCTATGGAGGCTTCTTATGG, and adk F: TTACTTGGACCTCCAGGTGC and adk R: GCAGCCTTAGGAAGTGGAAA. Equal amounts of DNA-free RNA (5 μg) was reverse transcribed to complementary DNA (cDNA) as follows. 20 μg RNA was incubated with 3 μg random hexamers and RNaseOUT ribonuclease inhibitor in a total volume of 16.4 μl, at 70°C for 10 min and then cooled on ice. For cDNA synthesis, 6 μl First Strand buffer, 0.6 μl dNTP mix (25 mM each dATP, dCTP, dGTP, dATP), 0.4 μl actinomycin D (1.2 mg/ml), 3 μl DTT (0.1 M) and 2 μl Superscript III were added to a total volume of 33 μl. Second strand cDNA synthesis was not performed in order to retain the strand specific sequence determination [70]. Samples were then incubated for 2 h at 42°C, following which RNA was hydrolysed with 1.5 μl NaOH (1 M) for 20 min at 70°C. Finally, samples were neutralised with 1.5 μl HCl (1 M) and cDNA was purified using a G50-Sephadex column (Sigma-Aldrich), according to manufacturer’s instructions. Samples were screened for the presence of cDNA using specific primer pairs (above).

### Library construction and sequencing

Libraries were constructed by shearing the purified cDNA using a Covaris LE220 focused ultrasonicator to give fragments in the range of 150–250 bp. This was followed by an end-repair incubation with T4 DNA polymerase, Klenow polymerase and T4 polynucleotide kinase (to phosphorylate blunt-ended fragments) for 30 min at 20°C. cDNA samples were then 3′ adenosine-tailed via the addition of Klenow exo- and dATP for 30 min at 37°C to reduce concatamerisation. Illumina adaptors (containing complementary sites to oligonucleotide anchors on the flow cell surface and primer sites for sequencing) were then ligated onto the cDNA repaired ends, and ligated fragments were electrophoretically separated from any unligated adapters based on size-selection. Fragments were then isolated via gel extraction. Libraries were amplified via PCR (18 cycles), quantified and denatured with 2 M NaOH to generate single stranded cDNA for sequencing. Samples were then loaded onto an Illumina flow cell to which the samples hybridise to the lawn of complementary oligonucleotide primers. Flow cell primers were then extended for 75 sequencing cycles, ultimately yielding clusters of clonally amplified cDNA templates. All steps were performed according to the manufacturer’s recommendations.

### RNASeq analysis

Transcripts were mapped using SMALT (http://www.sanger.ac.uk/resources/software/smalt/). Differential expression analysis was performed using R version 3.0.0 and DESeq statistical analysis package [71]. For comparison with proteomic data, moderated log-fold changes were calculated using the variance stabilizing transformation supplied by Deseq. *P*-values were corrected for multiple testing using the Benjamini–Hochberg method, and a q-value threshold of 0.01 was used to identify differentially regulated genes with an expected false discovery rate of 10% (see Additional file 1).

### Protein extraction and in-gel digestion

Three biological replicates of *C. difficile 630 wild-type*, *C. difficile 630Δerm* and *C. difficile 630ΔermΔspo0A* cultures were prepared. Briefly, ~10^10^ cells from exponentially growing *C. difficile* were harvested by centrifugation, resuspended in 300 μl lysis buffer (8 M urea, 2 M thiourea, 4% sodium dodecyl sulphate (SDS), 20 mM tris(2-carboxyethyl)phosphine (TCEP) in PBS) and incubated at 70°C for 10 min. Cells were then mechanically disrupted using acid-washed glass beads (size 425–600 μm; Sigma Aldrich) and a FastPrep ribolyser. The lysate supernatant was collected and clarified by centrifugation at 14,000 rpm for 30 min. Finally, samples were alkylated with a final concentration of 5 mM idoacetamide (IAA; Sigma-Aldrich). Samples with an equivalency to ~2 × 10^7^ cells were loaded to a 12% Bis-Tris NuPAGE gel (Invitrogen) for protein separation. 18 bands were excised from each sample lane followed by in-gel digestion with trypsin in 50 mM TEAB. Peptides were extracted by 50% acetonitrile/0.5% formic acid and dried in a SpeedVac. For one of the biological replicate, three technical replicates from the SDS-PAGE step were prepared such that were five replicates in total for proteomic analysis.

### Peptide dimethyl labelling and LC-MS/MS analysis

The dried peptides were derivatized with dimethyl triplex with cross-labelling using the standard in-solution protocol by Boersema [72], i.e. all primary amine (the N-terminus and the side chain of lysine residues) were converted to dimethylamines through reaction with formaldehyde (light), formaldehyde-D2 (intermediate) or formaldehyde-13C-D2 (heavy) and cyanoborohydride (light and intermediate) or cyanoborodeuteride (heavy).

The differentially labelled samples were mixed correspondingly before the nanoLC-MS/MS analysis on a LTQ Orbitrap Velos (Thermo Fisher) hybrid mass spectrometer equipped with a nanospray source, coupled with an Ultimate 3000 RSLCnano System (Dionex). The system was controlled by Xcalibur 2.1 (Thermo Fisher) and DCMSLink 2.08 (Dionex). Only 1/3 of total volume of each sample was submitted to analysis. Samples were first loaded and desalted on a PepMap C18 trap (0.3 mm id × 5 mm, 5 μm, Dionex) at 10 μL/min for 15 min, then peptides were separated on a 75 μm id × 50 cm PepMap RSLC column (Dionex, 2 μm) over a 120 min linear gradient of 4–32% CH_3_CN/0.1% FA at a flow rate at 300 nL/min. The LTQ Orbitrap Velos was operated in the “Top 10” data-dependant acquisition mode. The 10 most abundant and multiply-charged precursor ions in the MS survey scan in the Orbitrap (m/z 400 – 1500, with the lock mass at 445.120025) were dynamically selected for collision induced dissociation fragmentation (MS/MS) in the LTQ Velos ion trap. The ions must have a minimal signal above 2000 counts. The preview mode of FT master scan was disabled. The Orbitrap resolution was set at 60,000 at m/z 400 with one microscans. The isolation width for the precursor ion was set at 2 Th. The normalized collision energy was set at 35% with activation Q at 0.250 and activation time for 10 msec. The dynamic exclusion mass width was set at ±20 ppm and exclusion duration for 60 seconds. To achieve high mass accuracy, the AGC (Automatic Gain Control) were set at 1 × 10^6^ for the full MS survey in the Orbitrap with a maximum injection time at 100 msec, and 5000 for the MS/MS in the LTQ Velos with a maximum injection time at 300 msec.

### Protein identification and quantification

The raw files were processed with MaxQuant Software (version 1.3.0.5, http://maxquant.org) for protein identification and quantification. The Andromeda search engine was used to search the MS/MS spectra using the following parameters: trypsin/P with maximum 2 missed cleavages sites; peptide mass tolerance at first search was set at 20 ppm; MS/MS fragment mass tolerance at 0.49 Da, and top 6 MS/MS peaks per 100 Da and a minimum peptide length of 6 amino acids were required. The mass accuracy of the precursor ions was improved by the time-dependent recalibration algorithm of MaxQuant. Fixed modification for Carbamidomethyl and variable modifications for Deamidated (NQ) and Oxidation (M) were used, and a maximum of three labelled amino acids per peptide were allowed. The protein databases were extracted from annotated genome databases of *C. difficile 630 (June 2013)*, and the contaminant database was supplemented by MaxQuant.

False discovery rates (FDR) were estimated based on matches to reversed sequences in the concatenated target-decoy database, and an FDR threshold of 1% was used for proteins and peptides. Peptides were assigned to protein groups, a cluster of a leading protein(s) plus additional proteins matching to a subset of the same peptides. Protein groups with posterior error probability (PEP) values over 0.01 or matches to reversed database or contaminants were discarded. Protein identification was reported for protein groups with at least one unique peptide.

MaxQuant normalized protein ratios of the *C. difficile spo0A* mutant over the parental *C. difficile 630Δerm* strain were converted to Log2 values. For each sample with three technical replicates, the Log2 protein ratio used was derived from the mean value of three repeat analyses. The final Log2 protein ratios reported represents the mean values of three biological replicates, and only values with at least three ratio counts were used. The protein group ratios from three biological replicates were also analyzed in Perseus (version 1.3.0.4), using the Significance B method which considered both the protein ratio and intensities of the peptide ions. Protein groups that were positive in Significance B tests of at least two biological replicates are reported as significant.

### Transmission electron microscopy

Grids were prepared by briefly submerging slides into Formvar (0.1%) in dry chloroform. Formvar-carbon-coated support films were then floated onto distilled water, after which grids were placed onto the film before lifting onto parafilm and air-drying. Fresh bacterial colonies were picked, suspended in ammonium acetate and loaded onto the film side of the grid. An equal volume of ammonium molybdate (1%) was added to the film and immediately drained with filter paper. Samples were allowed to air-dry and were visualised via TEM as described above.

### Butyrate measurements

Culture supernatants of exponentially growing *C. difficile* 630∆*erm* strains were acidified, converted to *t*-butyldimethylsilyl derivatives as previously described and quantified by capillary gas chromatography [73]. Butyrate quantification was performed in triplicate.

### Availability of supporting data

The updated annotation for *Clostridium difficile* 630 described in this study is available in the main genomic sequence repositories under the accession number AM180355 (http://www.ebi.ac.uk/ena/data/view/am180355) and at http://www.sanger.ac.uk/resources/downloads/bacteria/clostridium-difficile.html. Fastq files from RNAseq analysis are available in ArrayExpress under the accession number E-ERAD-97 (http://www.ebi.ac.uk/arrayexpress/experiments/E-ERAD-97/).

## Abbreviations

Spo0A: Stage 0 sporulation protein A; erm: Erythromycin resistance; RNAseq: RNA sequencing; SDS-PAGE: Dodecyl sulfate polyacrylamide gel electrophoresis; FDR: False discovery rate; TEM: Transmission electron microscopy.

## Competing interests

The authors declare that they have no competing interests.

## Authors’ contributions

LJP, GD and TDL conceived the study. LJP, LY, DG and SHD performed lab work. LJP, HPB, LY, LB and MJM contributed to the bioinformatics analysis. HPB, LY, WKS, RPF, HJF, JSC and TDL analysed and interpreted the data. HPB, WKS, RPF, HJF, GD and TDL wrote the paper. All authors read and approved the final manuscript.

## Supplementary Material

Additional file 1 **Identification of differentially expressed genes in *C. difficile *** **630** ***∆erm spo0A *** **mutant by transcriptional profiling.** Scatter plot of the log_2_ fold changes against the normalised mean read abundance per gene (calculated at the base level). Red dots represent genes considered to be significantly differentially expressed (*P* = ≤ 0.01). Black dots signify genes not deemed to be significantly differentially expressed according to these criteria. Click here for file

Additional file 2 **Table summarizing genes differentially expressed in a *C. difficile *** **spo0A mutant relative to the parental strain based on transcriptomics and proteomics.** Datasets are presented in the context of the entire updated reference genome annotation for *C. difficile* 630. Click here for file

Additional file 3 **Table summarizing gene regulation genes that are controlled by *C. difficile *** **Spo0A.** Click here for file
