# Supplementary material for: Functional genomics reveals that Clostridium difficile Spo0A coordinates sporulation, virulence and metabolism
Source: BMC Genomics. 2014 Feb 25;15:160. doi: 10.1186/1471-2164-15-160 (PMC4028888; doi:10.1186/1471-2164-15-160)
Supplement: Additional file 1 — Identification of differentially expressed genes in C. difficile 630 ∆erm spo0A mutant by transcriptional profiling. Scatter plot of the log2 fold changes against the normalised mean read abundance per gene (calculated at the base level). Red dots represent genes considered to be significantly differentially expressed (P = ≤ 0.01). Black dots signify genes not deemed to be significantly differentially expressed according to these criteria. [file 1471-2164-15-160-S1.PDF]

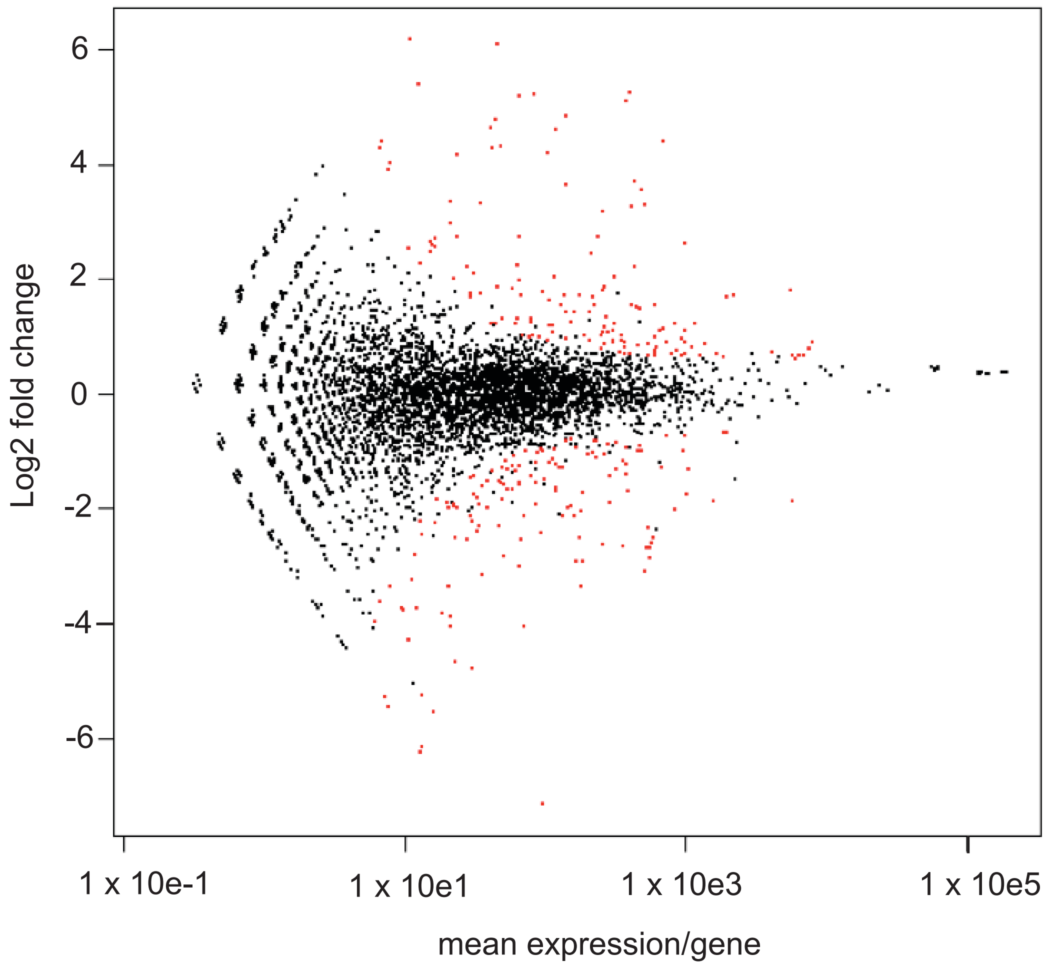

Additional file 1: Identification of differentially expressed genes in *C. difficile* 630 *spo0A* mutant by transcriptional profiling. Scatter plot of the log2 fold changes against the normalised mean read abundance per gene (calculated at the base level). Red dots represent genes considered to be significantly differentially expressed (P = ≤ 0.01). Black dots signify genes not deemed to be significantly differentially expressed according to these criteria.
