# Supplementary material for: Functional genomics reveals that Clostridium difficile Spo0A coordinates sporulation, virulence and metabolism
Source: BMC Genomics. 2014 Feb 25;15:160. doi: 10.1186/1471-2164-15-160 (PMC4028888; doi:10.1186/1471-2164-15-160)
Supplement: Additional file 3 — Table summarizing gene regulation genes that are controlled by C. difficile Spo0A. [file 1471-2164-15-160-S3.DOCX]

**Additional file 3.** Gene regulation genes that are controlled by *C. difficile* Spo0A.

| **Up-regulated in 630∆*spo0A*** | | | |
| --- | --- | --- | --- |
| Gene identifier | Class | Gene product | Associated protein domains/ families |
| CD1579 | 6.1.2 | Two-component sensor histidine kinase,sporulation-associated spo0A | PAS domain, Two-component regulatory system, GHKL domain |
| CD1064/ ccpA | 6.3.5 | Transcriptional regulator, LacI family | Transcription regulator HTH, LacI, Periplasmic binding protein |
| CD2711 | 6.3.6 | Transcriptional regulator, LysR family | Transcription regulator HTH- LysR, substrate-binding- LysR |
| CD0616 | 6.3.12 | Transcriptional regulator, MerR family | HTH, transcription activator, effector binding |
| CD2578/ stk | 6.4.1 | putative serine/threonine-protein kinase and phosphatase | Protein kinase, catalytic domain, Serine/threonine- / dual specificity protein kinase, PASTA |
| CD0205 | 6.5.0 | Transcription antiterminator, PTS operon regulator | PTS system, M trans-acting positive regulator, PRD |
| CD0283 | 6.5.0 | Transcription antiterminator, PTS operon regulator | Sigma factor 54, AAA+ ATPase, PTS system |
| CD0292 | 6.5.0 | Transcriptional regulator, HTH-type | HTH, Lambda repressor-like, DNA-binding |
| CD0618 | 6.5.0 | Transcriptional regulator, LytR family | LytTR DNA-binding |
| CD1494 | 6.5.0 | Transcriptional regulator, HTH-type | HTH, Lambda repressor-like, RmlC-like cupin |
| CD1893 | 6.5.0 | putative oligonucleotide binding regulator | YbaK/aminoacyl-tRNA synthetase-associated |
| CD2134 | 6.5.0 | putative signaling protein | GGDEF, Adenylyl cyclase class-3/4/guanylyl cyclase, Adenylyl cyclase class-3/4/guanylyl cyclase |
| CD2214/ SinR | 6.5.0 | Transcriptional regulator, HTH-type | HTH, Lambda repressor-like |
| CD2215 | 6.5.0 | Transcriptional regulator, HTH-type | HTH, Lambda repressor-like |
| CD2765 | 6.5.0 | Transcriptional regulator, LytR family | Cell envelope-related transcriptional attenuator |
| CD3133 | 6.5.0 | Transcription antiterminator, PTS operon regulator | Leucine-rich repeat, PTS system, M trans-acting positive regulator |
| CD3158 | 6.5.0 | Transcriptional regulator, TRAP family | Tryptophan RNA-binding attenuator protein-like |
| CD3166 | 6.5.0 | Transcriptional regulator, hxlR family | HTH- hxlR type, Winged HTH |
| **Down-regulated in 630∆*spo0A*** | | | |
| Gene identifier | Class | Gene product | Associated protein domains/ families |
| CD0770/ spoIIAA | 6.1.2 | Anti-sigma F factor antagonist | STAS, Anti-sigma factor antagonist |
| CD0771/ spoIIAB | 6.1.2 | Anti-sigma F factor (Stage II sporulation protein AB) | Histidine kinase-like ATPase, Anti-sigma F factor |
| CD0820 | 6.1.2 | Two-component response regulator | Signal transduction response regulator, CheY-like |
| CD1492 | 6.1.2 | Two-component sensor histidine kinase,sporulation-associated spo0A | PAS domain, Histidine kinase-like ATPase, Signal transduction histidine kinase |
| CD0772/ sigF | 6.2.1 | RNA polymerase sigma-F factor | RNA polymerase sigma-70 regions |
| CD1498/ sigA2 | 6.2.1 | RNA polymerase sigma factor SigA2 (sigma-43) | RNA polymerase sigma-70 regions |
| CD2642/ sigG | 6.2.1 | RNA polymerase sigma-G factor | RNA polymerase sigma-70 regions |
| CD2643/ sigE | 6.2.1 | RNA polymerase sigma-E factor | RNA polymerase sigma-70 regions |
| CD1883 | 6.3.2 | Transcriptional regulator, AraC family | Homeodomain-like |
| CD1996 | 6.3.2 | Transcriptional regulator, AraC family | transcription activator, effector binding, Regulatory factor |
| CD2665 | 6.3.2 | Transcriptional regulator, AraC family | Transcription regulator HTH, Homeodomain-like, RmlC-like jelly roll fold |
| CD1079 | 6.3.6 | Transcriptional regulator, LysR family | Transcription regulator HTH , LysR, substrate-binding |
| CD0390/ bglG | 6.5.0 | Transcription antiterminator, PTS operon regulator | CAT RNA-binding, PRD |
| CD1388 | 6.5.0 | putative trancriptional regulator | HTH |
| CD2445 | 6.5.0 | putative transmembrane signaling protein,TspO/MBR family | TspO/MBR-related |
| CD2668 | 6.5.0 | Transcription antiterminator, licT family | CAT RNA-binding, PRD |
| CD3117/ bglG2 | 6.5.0 | Transcription antiterminator, PTS operon regulator, bglG2 | CAT RNA-binding, PRD |
| CD3138/ bglG4 | 6.5.0 | Transcription antiterminator, PTS operon regulator, bglG4 | CAT RNA-binding, PRD |
| CD3175/ cggR | 6.5.0 | Transcriptional regulator, SorC family | Putative sugar-binding domain, winged HTH |
